# The Pay System and the Profession

**Published:** 1894-11-10

**Authors:** 


### The Hospital

An Institutional Journal of

The Medical Sciences and Hospital Administration, with

AN EXTRA NURSING SUPPLEMENT.

Vol. XVII.—No. 424.] [Nov. 10, 1894.

### THE PAY SYSTEM AND THE PROFESSION.

A few years ago the Great Northern Hospital amalgamated with the Committee formed in the North of London to establish a central hospital there for the benefit of the poorer population. The new hospital was entitled the Great Northern Central Hospital, a site was ultimately purchased at Islington, and upon it one of the most complete hospital buildings in the metropolis has been erected and opened. Several public meetings were held when the new Central Hospital was incepted, and after the amalgamation of the two institutions had taken place. It was clearly laid down at the meetings that a new departure would be made by the management, and that arrangements would be made in the new buildings for the admission and treatment of various grades of paying patients. It was rightly felt by those most interested in the scheme that to establish one more pauperising agency for the demoralisation of the inhabitants of North London, as a result of the public spirited efforts of the representative leaders of the people in that district, would show little knowledge and less sense. It has been said, and said with force and truth, that the great out-patient departments of the voluntary hospitals in this country do more to tempt the poorer classes of this country with a spirit of pauperism than anything else known to our generation. At the same time the Great Northern Central Hospital authorities recognised that the doors of their institution must be freely opened to the deserving poor at all hours without payment or hindrance of any kind. There was no secret whatever about the proposals to which we refer; they commended themselves to the judgment of the inhabitants of the district in which the new hospital was to be situated; and, in the result, large sums of money have been subscribed, and the new hospital has been completed in its entirety, and is now open for the reception of patients. The principle of the admission of patients laid down was that, whereas every person who could show a good title was to be treated as a free patient, provision was to be made by which it would be possible to grant to every patient the privilege of paying something, however small, for the relief he received according to his means. It was clearly understood that, in addition to the pay-patients, who would give sums varying, say, from five shillings to two guineas a week, a separate block was to be set aside for the reception of paying patients of a higher class, who were to pay in proportion to the accommodation and benefits which they received, so as to entail no loss upon the hospital, and, in certain circumstances, to yield a profit to the institution.

It is essential that the foregoing facts be borne in mind to enable the profession and the public to rightly judge the action of certain members of the medical profession practising in the district, a deputation of whom had an interview with the committee of the hospital on the 19th ultimo. This deputation presented a petition signed by 204 medical practitioners in the North of London, which declared in effect that it is at all times inexpedient for a hospital to attempt to make money, and an injustice to the subscribers that others than the necessitous poor should receive the benefits of such an institution; that the opening of pay wards in a public hospital was a needless interference with medical practice, and detrimental to the interests both of medical men and nurses conducting nursing homes in the district; that it is almost quite certain that the existing abuse of hospital relief will be immeasurably increased by the institution of pay wards; that the medical and surgical staff of a hospital, who attend to pay patients gratuitously, inflict a proportionate pecuniary injury upon the medical practitioners in the neighbourhood of the hospital. The chief objection urged by the various speakers to the institution of pay wards, in addition to those already stated, was that every pay patient admitted presumably excluded a person who could not pay, and that the medical staff of other hospitals declined to treat such patients. In reply to the deputation, the chairman of the committee stated that the scale of payments had been so arranged as to simply cover the expenses, there was no idea of making a profit; that, as the certificate of each applicant had to be signed by the medical attendant, the medical profession had it in their power to prevent unsuitable persons from being treated in the pay wards.

It will be seen that the deputation's objections are none of them fundamental, and that all of them are capable of being removed by a code of adequate rules, carefully prepared and strictly enforced. We are convinced however, that the committee of the Great

Northern Central Hospital ought to have the support not only of the public but of the medical profession in the new departure they have initiated. If every voluntary hospital declined to admit any patient under any circumstances as a free patient unless proof was forthcoming, either before admission, or, in a case of accident, within a week of the date of admission, that the patient was entitled to claim free medical relief, the present abuse of the hospitals would soon be a thing of the past. We would, therefore, urge the committee of the Great Northern Central Hospital to nominate a small special committee of medical men and laymen to prepare a code of rules for the admission of pay patients. Those rules should grant permission to each private practitioner to follow his patient into the pay wards, and to attend him there at his option according to the scale of fees embodied in the rules. To enable this to be done a line must be drawn between pay patients in the general wards, who in no case would pay more than, say, two guineas a week, and who would be entitled to have, like the free cases, all the advantages the hospital can afford. Patients paying from two guineas per week and upwards should be treated in wards set apart entirely for the accommodation of pay .patients Twenty-five per cent. of all moneys received from the patients in the pay wards should be placed to a separate fund, to be devoted entirely to the payment of the medical attendant in charge of each case, whether that attendant was a member of the hospital staff, or a practitioner unattached to the hospital. In this way every patient who entered the Great Northern Hospital would have the opportunity of maintaining his self-respect, whilst he was saved from any risk of being pauperised by hospital treatment. The medical profession would, at the same time, be protected from the loss which it now experiences, owing to the large number of patients who obtain free relief at the hospitals, when they can afford to pay a private practitioner. Under such a system as that here proposed justice would be done to the subscribers, to the profession, and to the patients, as well as to the institution itself. There would be no interference with medical practice, and the medical and surgical staff of the hospital, especially its junior members, would be relieved of the injustice attending the present system of unlimited free medical relief. By placing the burden of proof upon the patients we should secure at least one hospital in London where hospital abuse would soon cease to exist. For these and other reasons we are confident that the wise example thus set by the Great Northern Central Hospital would soon find favour with every similar institution, and that reform would be introduced into the present system of hospital relief which must prove of momentous importance, not only to the public, the patients, and the hospitals, but to the whole body of the medical profession. We hope, therefore, to hear that such a special committee as we have suggested has been nominated, and then we have little doubt that before many months are past, the new system will have won for itself a popularity which will ensure its permanent success.

